# Relationship between Perceived Stress, Obesity, and Hypertension in Korean Adults and Older Adults

**DOI:** 10.3390/healthcare11162271

**Published:** 2023-08-11

**Authors:** Sung-Eun Park, Wi-Young So, Yun-Sun Kang, Jong-Hyun Yang

**Affiliations:** 1 Department of Sports Science, Korea Institute of Sport Science, Seoul 01794, Republic of Korea; separk@kspo.or.kr; 2 Sport Medicine Major, College of Humanities and Arts, Korea National University of Transportation, Chungju-si 27469, Republic of Korea; wowso@ut.ac.kr; 3 Department of Physical Education, Graduate School of Education, Sogang University, Seoul 04107, Republic of Korea; 4 Goyang Dance Company, Professional Arts Organization, Goyang-si 10417, Republic of Korea; 5 Department of Physical Education, Incheon National University, Incheon 22012, Republic of Korea

**Keywords:** body mass index, high blood pressure, overweight, strain, tension

## Abstract

Background: Perceived stress has a significant effect on metabolic diseases, including obesity and hypertension. However, the association between stress levels, obesity, and hypertension according to age and sex is not fully understood. Therefore, this study investigated the relationship between stress levels and obesity and hypertension in Korean adults and older adults. Methods: We analyzed data from the 2015 survey of the Korea National Physical Fitness Project conducted by the Korea Institute of Sports Science and the Korea Ministry of Culture, Sports, and Tourism. Of the 3457 participants, 2829 were adults (20–64 years old) and 628 were older adults (≥65 years old). The correlation between obesity and hypertension according to the degree of perceived stress (low, medium, and high) was analyzed using the chi-square test. Binary logistic regression analysis was used to investigate the influence of perceived stress levels on obesity and hypertension. Age, body mass index (BMI), blood pressure, exercise frequency, smoking, breakfast, and sleeping hours were included as covariates. Results: In adult males, perceived stress levels, age, and diastolic blood pressure were found to have a significant impact on obesity rates, whereas age and breakfast had a significant effect on hypertension rates. In adult females, age and systolic blood pressure were found to significantly influence obesity rates, whereas age, BMI, and exercise frequency had a significant impact on hypertension rates. In older adult females, perceived stress levels and systolic blood pressure were found to significantly impact obesity rates, and sleep duration influenced the rates of hypertension. The effect of perceived stress level on obesity and hypertension rates was less pronounced in the elderly population than in the adult population. Conclusions: This study revealed age and sex differences in the relationship between perceived stress, obesity, and hypertension among Koreans. These findings contribute to a better understanding of the complex relationship between perceived stress and metabolic disorders and emphasize the need for a deeper understanding of the specific factors involved in the prevention and management of metabolic diseases.

## 1. Introduction

Stress is a significant factor that affects various aspects of physical and mental health in modern society. Excessive stress can lead to negative physiological changes, such as dysregulation of the autonomic nervous system and the hypothalamic–pituitary–adrenal axis. Particularly, “perceived stress”, which is an individual’s subjective assessment of the level of stress they are experiencing over a period, can be shaped by one’s life experiences, physical and mental health conditions, and social support, among others [1,2,3]. High levels of perceived stress might result in less engagement in physical activity and a greater likelihood of indulging in unhealthy behaviors such as poor dietary habits and smoking [4], and have implications for various health issues, including compromised immune function [5,6,7]. If such stress persists chronically, it can disrupt the body’s equilibrium and lead to various conditions such as cardiovascular diseases and diabetes, as well as mental disorders like depression and anxiety [8,9,10]. Conversely, individuals experiencing high levels of stress may increase their activities such as exercise as a coping mechanism for stress. Evidence from systematic reviews regarding the relationship between psychological stress and physical activity has been reported, with some prospective studies suggesting that several active coping mechanisms such as exercise may be employed in response to perceived stress [11].

Perceived stress is also associated with obesity and hypertension [12]. Increased secretion of the stress hormone cortisol plays a role in energy provision for the body, but can trigger increased appetite and weight gain in the long term [13]. Continuous exposure to perceived stress can lead to adverse behaviors, such as unhealthy dietary patterns, which may act as a risk factor for obesity [14]. Contrarily, a study involving college students reported a stress-eating paradox, showing divergent responses: while some exhibited reduced food intake upon exposure to cognitive stress, others showed increased body weight [15,16,17]. Moreover, eating habits in response to perceived stress can differ based on their characteristics (e.g., age, sex, ethnicity, etc.) [18,19]. Therefore, stress does not inevitably result in weight gain or obesity. The impact of stress on obesity may vary depending on an individual’s sex and age, as well as their perception of stress, coping mechanisms, and control over stressful situations [20]. These factors may vary greatly depending on individual circumstances.

Stress and physiological stress responses directly affect the cardiovascular system. During stressful situations, hemodynamic changes are induced to meet increased metabolic demands and maintain homeostasis. As a result of vasodilation due to the activation of sympathetic nerves in vascular smooth muscle cells and increased contractility by cardiac myocytes, blood pressure may temporarily increase [21]. This transient increase in blood pressure is a natural and necessary response to stress in the short term. However, if these temporary increases occur repeatedly, they may become chronic and contribute to the development of long-term hypertension [22,23,24]. Activation of the autonomic nervous system due to acute stress stimulates the secretion of stress hormones, such as cortisol and adrenaline, increasing heart rate and blood pressure [25]. Repeated exposure to such stress can lead to long-term changes in the systems regulating heart rate and blood pressure, eventually resulting in hypertension [26]. As individuals age, changes occur in their metabolism. The loss of lean muscle mass results in a reduction in basal metabolic rate (BMR), which is the minimum amount of energy needed for survival. Increases in fat mass can contribute to weight gain and an elevated risk of metabolic diseases such as diabetes [27]. Aging can also decrease physical capabilities, such as strength, and increase the risk of falls, thereby escalating exposure to physical activity injuries [28]. Furthermore, while older individuals may be psychologically healthy, the incidence of mental illnesses like depression and anxiety might increase with age, and they may be exposed to psychosocial stressors such as changes in social roles, health problems, and bereavement [29]. Cognitive functions related to learning and memorizing new content may decline in older adults; however, the mechanisms for assessing and coping with stress tend to improve due to experiences and changes in socioeconomic and psychosocial roles that accompany aging [30,31]. Older adults are often better at regulating their emotions than younger adults, leading to positive effects such as enhanced emotional well-being [32]. Adults and older adults exhibit significant physical and psychological differences. Understanding these age-related differences is crucial, as they can affect disease risk, prevention, and treatment strategies.

As such, perceived stress, well known to greatly impact health in modern society, stands as one of the major risk factors for chronic diseases, including obesity and hypertension. In Korea specifically, more than half of the population reports experiencing stress in their daily lives, indicating a relatively high stress sensitivity level compared to other Organization for Economic Co-operation and Development countries [33]. This suggests that perceived stress might contribute to the increasing prevalence of chronic diseases like obesity and hypertension among Koreans. However, the relationship between perceived stress, obesity, and hypertension in adults and older adults remains inadequately understood. In particular, with the older adult population in Korea anticipated to surge from 17.5% in 2022 to 46.4% in 2067 [34], the country is experiencing rapid aging. This makes it more crucial to comprehend this relationship among older adults. Therefore, we sought to deepen our understanding of the interplay between perceived stress, obesity, and hypertension in adults and older adults. To this end, a large sample of Korean adults and older adults was used to statistically analyze the relationships among these factors and assess how they differ by age and sex. Such analyses could contribute to the formulation of appropriate strategies to address obesity and hypertension in the future.

## 2. Materials and Methods

### 2.1. Participants

In this study, we analyzed data from the 2015 survey of the Korea National Physical Fitness Project, a biennial survey conducted by the Korea Institute of Sport Science and the Korea Ministry of Culture, Sports, and Tourism. This survey strategically selects samples based on sex, age, and region, and covers the entirety of the Republic of Korea. Participants whose data were missing from the stress perception survey were excluded from our study. A total of 3457 participants were included in the study, which comprised 2829 adults (20–64 years old) and 628 older adults (≥65 years). The baseline characteristics of the adults and older adults are presented in Table 1, and the physical and anthropometric characteristics of the participants are presented in Table 2. All research procedures complied with the principles of the Declaration of Helsinki and since data sets on the raw data from the Institute of Sports Science and the Korea Ministry of Culture, Sports, and Tourism did not include private identifier information, such as name, telephone numbers, home addresses, and social security numbers, ethical approval was not required.

### 2.2. Perceived Stress

To assess perceived stress levels, the participants were asked to rate their average day-to-day cognitive stress using the following questions, assessed according to a self-reported response on a 5-point scale.

- Question: What is the average daily level of stress experienced?
- Possible responses: Very low, low, moderate, high, very high.

In the responses, those who answered “very severe” or “severe” were classified as the high perceived stress group, those who answered ”moderate” were classified as the moderate perceived stress group, and those who answered “slight” or “very slight” were classified as the low perceived stress group. The validity of these questions was confirmed by the Korea Institute of Sports Science and the Korea Ministry of Culture, Sports, and Tourism [35].

### 2.3. Obesity

Body mass index (BMI), a measure obtained by dividing a person’s weight in kilograms by the square of their height in meters, is a widely used indicator that shows a high correlation with body fat content and is often used to evaluate obesity. The World Health Organization (WHO) defines individuals with a BMI of 25 kg/m^2^ or higher as overweight, and those with a BMI of 30 kg/m^2^ or higher as obese. However, for the Asia-Pacific region, the WHO and the Korean Society for the Study of Obesity [36] define individuals with a BMI of 23 kg/m^2^ or higher as overweight, and those with a BMI of 25 kg/m^2^ or higher as obese. In this study, we adopted the latter standard, classifying participants with a BMI of 25 kg/m^2^ or higher as obese. This definition aligns with regional standards and thus allows for a more accurate assessment and comparison within the target population.

### 2.4. Hypertension

The participants rested by sitting for at least 10 min prior to the blood pressure measurement. Thereafter, participants were seated with their arms resting on a table, ensuring that the brachial artery and the heart were at the same height. A nurse measured systolic and diastolic blood pressure (SBP and DBP) using a mercury sphygmomanometer (ALPK, Tokyo, Japan) and a stethoscope. Blood pressure was measured twice at 1–2 min intervals, and the average value was recorded. If the blood pressure was high, it was measured three times, and the average of the second and third measurements was recorded as the final value [37].

In accordance with the criteria established by the American Heart Association [38] and the Korean Society of Hypertension [37], individuals are considered to have normal blood pressure when their systolic blood pressure (SBP) is <120 mmHg and their diastolic blood pressure (DBP) is <80 mmHg. Prehypertension is defined as an SBP of 120–139 mmHg or a DBP of 80–89 mmHg. Hypertension is classified as an SBP of ≥140 mmHg or a DBP of ≥90 mmHg. Moreover, hypertension is further categorized as stage 1 hypertension, defined by an SBP of 140–159 mmHg or a DBP of 90–99 mmHg, and stage 2 hypertension, defined as an SBP of ≥160 mmHg or a DBP of ≥100 mmHg. We used these criteria to classify participants with hypertension in our study. This classification is consistent with the accepted guidelines and facilitates a more accurate assessment of the population under investigation.

### 2.5. Exercise Frequency, Smoking, Breakfast, and Sleeping Hours

The relationship between perceived stress, obesity, and hypertension was analyzed considering factors that could potentially impact perceived stress, such as exercise frequency, smoking, breakfast habits, and sleep duration. Participants were asked to report the number of weekly workouts in response to the question, “How often do you exercise in a way that makes you sweat for at least 30 min per week?” Furthermore, when asked about smoking habits, participants were prompted to choose from the following options: “I currently smoke”, “I used to smoke, but I don’t now”, and “I have never smoked.” In this study, only those who indicated that they were current smokers were classified as such, excluding those who smoked but were currently non-smokers. Regarding breakfast habits, participants were asked whether they ate breakfast regularly and were given the options to answer, “I regularly eat breakfast”, “I irregularly eat breakfast”, “I skip breakfast every morning”, “I replace it with snacks”, and “Other”. Participants who reported eating breakfast either regularly or irregularly were categorized as breakfast eaters, whereas those who responded otherwise were categorized as non-breakfast eaters. Finally, participants were asked to report their average daily sleep duration in response to the question, “How many hours do you sleep per day on average?”

### 2.6. Procedure and Statistical Analysis

Data were presented as mean ± standard deviation or number (%). The independent *t*-test was used to compare continuous variables according to sex, and the chi-square test was used to compare categorical variables according to sex. The correlation between obesity and hypertension according to the degree of perceived stress (low, moderate, and high) was analyzed using the chi-square test. Binary logistic regression analysis was used to investigate the influence of perceived stress levels on obesity and hypertension. Age, SBP, and DBP were added as covariates in the logistic regression analysis for obesity, whereas age and BMI were included as covariates in the logistic regression analysis for hypertension. All data were analyzed using SPSS version 25.0 (IBM Corp., Armonk, NY, USA), and the statistical significance level was set at *p* < 0.05.

## 3. Results

### 3.1. Participant Characteristics

Regarding the baseline characteristics of adults and older adults (Table 1), both height and weight (*p* < 0.001) and DBP (*p* = 0.009) were higher in adults than in older adults. BMI was higher in older adults than in adults (*p* < 0.001), and SBP was similar between adults (123.97 ± 11.72 mmHg) and older adults (124.65 ± 11.52 mmHg). Among the perceived stress levels surveyed, low-level stress perception was higher in older adults than in adults, and medium- and high-level stress perception were higher in adults than in older adults (*p* < 0.001). The frequency of exercise was higher in older adults than in adults (*p* < 0.001), and the rate of smoking was higher in adults than in older adults (*p* < 0.001).

Regarding the physical characteristics of the participants (Table 2), the average age was 38.17 ± 12.71 years for adult males, 39.39 ± 13.09 years for adult females, 72.19 ± 5.25 years for older adult males, and 73.60 ± 5.87 years for older adult females. Height, weight, and BMI were higher in males than in females. Among adults, the obesity rate was higher in males (38.1%) than in females (16.0%) (*p* < 0.001), whereas it was higher in females (36.2%) than in males (32.3%) among older adults (*p* = 0.004). SBP and DBP were higher in males than in females among adults (*p* < 0.001), whereas among the older adults, SBP was higher in males (125.97 ± 12.01 mmHg) than in females (123.69 ± 11.07 mmHg) (*p* = 0.014), but DBP was similar between males (74.98 ± 9.45 mmHg) and females (75.40 ± 9.18 mmHg). The hypertension rate was higher in males than in females among both adults and older adults, and a significant difference was observed only in adults (*p* < 0.001). Among the surveyed perceived stress levels, the low and moderate perceived stress levels were higher in adult females than in males, and the high perceived stress levels were higher in adult males than in females; however, there was no significant difference. Among older adults, low perceived stress levels were higher in males than in females, and medium and high perceived stress levels were higher in females than in males; however, again, no significant differences were observed.

### 3.2. Correlation between Perceived Stress Levels and Obesity and Hypertension

In adult males, obesity was significantly correlated with perceived stress levels (*p* = 0.011). The proportion of obese individuals increased from 30.6% at low perceived stress levels to 39.9% and 39.6% at moderate and high perceived stress levels, respectively. However, there was no significant correlation between perceived stress and hypertension (*p* = 0.399) in adult males, or between perceived stress and obesity (*p* = 0.477) or hypertension (*p* = 0.750) in adult females. The proportions of obesity and hypertension were consistent across all perceived stress levels (Table 3). In older adult males, no significant correlation was found between perceived stress levels and obesity (*p* = 0.656) or hypertension (*p* = 0.857). The proportion of hypertension increased with perceived stress levels, but not significantly, and the proportion of obesity showed inconsistent changes according to perceived stress levels. In older adult females, there was no significant correlation between perceived stress levels and obesity (*p* = 0.062) or hypertension (*p* = 0.371). The proportion of obesity was slightly lower at moderate perceived stress levels, and the proportion of hypertension was slightly higher at high perceived stress levels; however, the difference was not significant (Table 4). These results suggest that the proportions of obesity and hypertension are higher in adult males than in females at higher perceived stress levels. Moreover, in both older adult males and females, there were no significant correlations between the proportions of obesity, hypertension, and perceived stress levels. In summary, these results suggest sex and age differences in the effects of perceived stress on obesity and hypertension.

### 3.3. Effects of Perceived Stress Levels on Obesity and Hypertension

**Figure 1:**
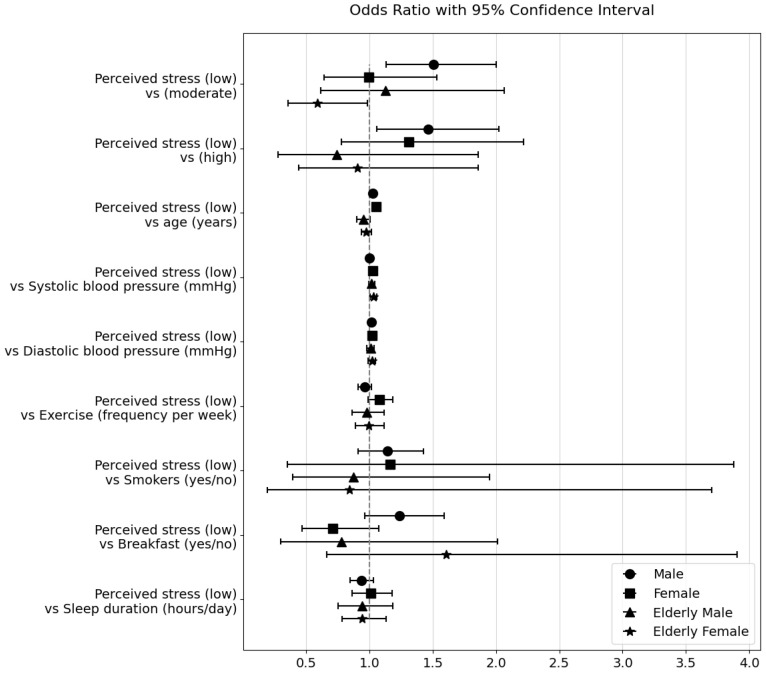
Odds ratio plot: Impact of perceived stress levels on obesity in Korean adults and older adults.

**Figure 2:**
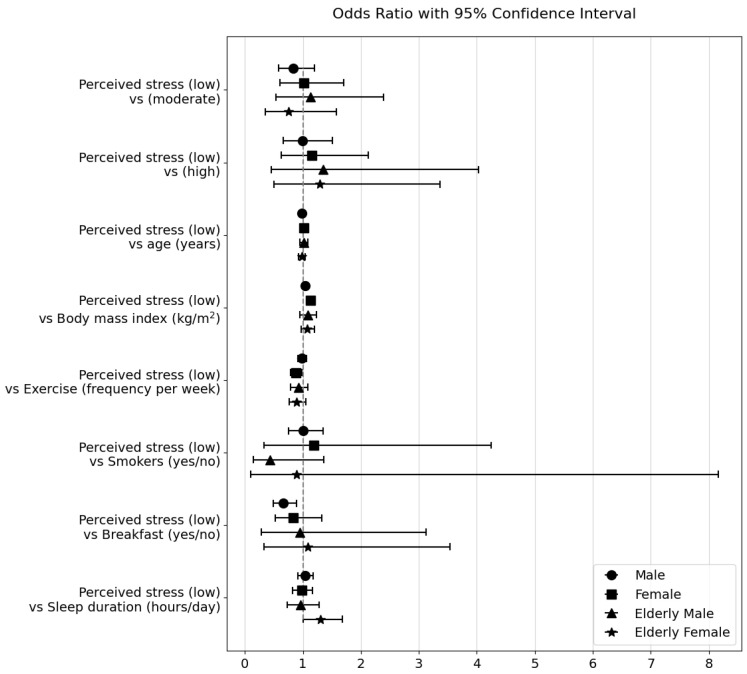
Odds ratio plot: Impact of perceived stress levels on hypertension in Korean adults and older adults.

The effects of perceived stress levels on obesity and hypertension are presented in Table 5 and Table 6 as well as Figure 1 and Figure 2, respectively. In adult males, after adjusting for age, blood pressure, frequency of exercise, smoking, breakfast, and sleeping hours, the odds ratio of obesity was 1.506 times higher at moderate perceived stress levels than at low perceived stress levels (95% confidence interval (CI), 1.132–2.002, *p* = 0.005) and 1.461 times higher (95% CI, 1.055–2.023, *p* = 0.023) at high perceived stress levels. However, in adult males, perceived stress levels did not significantly affect the rate of hypertension. Conversely, perceived stress levels did not significantly influence obesity or hypertension rates in adult females. Regarding the effect of age, for each one-year increase in age, the odds ratio of obesity increased 1.024 times (95% CI, 1.016–1.032, *p* < 0.001) in adult males. On the other hand, for each one-year increase in age, the odds ratio of hypertension decreased by 0.988 times (95% CI, 0.977–0.999, *p* = 0.030). In adult females, for each one-year increase in age, the odds ratio of obesity increased 1.049 times (95% CI, 1.034–1.064, *p* < 0.001), and the odds ratio of hypertension increased 1.020 times (95% CI, 1.003–1.038, *p* = 0.019). Regarding the effect of blood pressure, in adult males, for each one-unit increase in diastolic blood pressure, the odds ratio of obesity increased 1.015 times (95% CI, 1.001–1.029, *p* = 0.031), but systolic blood pressure did not significantly affect it. In adult females, for each one-unit increase in systolic blood pressure, the odds ratio of obesity increased 1.026 times (95% CI, 1.010–1.043, *p* = 0.002), but diastolic blood pressure had an odds ratio of 1.021 for obesity, indicating borderline significance but not a definitive effect (*p* = 0.055). Regarding the influence of BMI, for each one-unit increase in BMI, the odds ratio of hypertension increased 1.134 times (95% CI, 1.063–1.210, *p* < 0.001) in adult females, but there was no significant effect in adult males. As for the impact of exercise frequency, in adult females, for each one-unit increase in exercise frequency, the odds ratio of hypertension was reduced 0.878 times (95% CI, 0.787–0.981, *p* = 0.021). However, no significant effects were observed for the exercise frequency. In addition, no significant effects were found on the impact of smoking. Regarding the impact of breakfast, in adult males, eating breakfast reduced the odds ratio of hypertension by 0.660 times (95% CI, 0.487–0.893, *p* = 0.007). No other significant effects were found for breakfast, and no significant effects were identified in relation to sleep duration.

In older men, perceived stress levels did not significantly affect the rates of obesity or hypertension, even after adjusting for factors such as age, blood pressure, exercise frequency, smoking, breakfast, and sleeping hours. Age, blood pressure, BMI, exercise frequency, smoking, breakfast consumption, and sleeping hours did not significantly influence the rates of obesity or hypertension. In contrast, in elderly women, the odds ratio for obesity was 0.604 times lower at moderate cognitive stress levels than at low cognitive stress levels (95% CI, 0.353–0.982, *p* = 0.042). When considering the impact of blood pressure, each one-unit increase in systolic blood pressure (SBP) increased the odds ratio of obesity rates in elderly women by a factor of 1.029 (95% CI, 1.005–1.054, *p* = 0.017). Additionally, for every one-unit increase in sleeping hours, the odds ratio of the hypertension rate in elderly women increased by 1.303 times (95% CI, 1.011–1.680, *p* = 0.041), and the odds ratio of the obesity rate was 0.939, indicating borderline significance, but no definitive effect was observed. Other factors did not significantly affect the obesity or hypertension rates.

These results suggest that perceived stress levels have different impacts on obesity and hypertension rates in adults and older adults, depending on sex. Specifically, perceived stress had a more significant impact on obesity rates in adult males than in females, and diastolic blood pressure was also found to have an effect. In adult women, systolic blood pressure had a greater influence on obesity rates, and BMI and exercise frequency had a more significant impact on hypertension rates than perceived stress. The effects of the variables considered in conjunction with perceived stress levels on obesity and hypertension were more pronounced in adults than in older adults. In the geriatric population, a significant effect was found only in elderly women, where moderate perceived stress had a greater impact on obesity rates than men, and systolic blood pressure also had an influence. The effect of sleep duration on obesity and hypertension rates in older women is also noteworthy. Importantly, age had an impact on both obesity and hypertension rates in the adult population but not in the elderly population.

## 4. Discussion

This study discovered a significant relationship between perceived stress and obesity rates in adult males, as well as a notable connection between breakfast consumption and rates of high blood pressure. It also identified links between physiological factors, such as blood pressure, BMI, exercise frequency, and rates of obesity and hypertension in adult women. Interestingly, the effects of age on obesity and hypertension rates were observed only in adults. In older women, significant relationships were found between perceived stress, systolic blood pressure, and obesity rates, and proposed associations were suggested between sleep duration and rates of both obesity and hypertension. However, this study emphasizes the importance of exploring additional factors, bidirectional relationships, and potential mechanisms for a better understanding of these relationships.

The findings of this study align with those of previous research, indicating a stronger influence of perceived stress levels on obesity rates in adult males compared to females [14,39]. This is consistent with studies showing that hormonal and psychological factors contribute to sex differences in stress responses [40,41]. Chronic stress is often recognized as a factor that can lead to behavioral changes, such as eating habits, potentially contributing to weight gain [18]. Unhealthy eating habits, such as overeating or irregular eating, may serve as mechanisms through which perceived stress affects BMI and increases the risk of obesity [42,43,44]. However, it should be acknowledged that weight gain can also occur due to endocrine disturbances and dysregulation of the hypothalamic–pituitary–adrenal axis, regardless of caloric excess or deficit [45]. The findings of this study underscore the association between perceived stress levels and obesity in adult males, suggesting that males may be more vulnerable to obesity risk due to perceived stress. This highlights the need for a deeper understanding of sex differences in the relationship between perceived stress and obesity, as it can significantly impact approaches to addressing obesity.

The relationship between perceived stress and obesity is complex and influenced by various factors, including biological responses, personal coping, and social support [1,2]. However, it should be noted that this study did not directly measure lifestyle habits such as stress-related eating, which limits the ability to draw definitive conclusions from the observed associations. It should be understood that stress itself is a behavioral factor and pattern, and lacks a physiological basis and motivation. In addition, cortisol levels induced by stress and activity of the hypothalamic–pituitary–adrenal axis may lead to metabolic abnormalities. However, these factors may disrupt central fat accumulation irrespective of overt dietary changes [18,40,46]. These neuroendocrine pathways may vary with age and stress exposure. For instance, in older adults, retirement could result in reduced workload, and better coping mechanisms, honed through years of experience, might play a more effective role in regulating these pathways [30]. Future studies should consider comprehensive assessments of lifestyle behavioral factors, including eating habits, and physiological and psychological factors to better elucidate the mechanisms underlying the link between perceived stress and obesity.

Sex differences in perceived stress can be attributed to a variety of factors, including coping strategies, willingness to truthfully report stressful experiences, and hormonal influences. Previous research has indicated that men tend to utilize problem-focused coping while women typically employ emotion-focused coping [47]. It has been suggested that men may have difficulties accepting and expressing their emotions, while women may find it more challenging to take proactive measures in problem solving, although these patterns may vary under certain circumstances [48]. Additionally, women generally tend to report stress experiences more readily than men, and allostatic load and cardiometabolic profiles, which refer to the body’s ability to physiologically adapt to stressors, are typically much worse in men than in women [49]. Another important aspect to consider is the hormonal differences. Additionally, women possess hormonal abilities to regulate stress responses more efficiently than men, with estrogen playing a role in maintaining and enhancing frontal lobe function and neuronal viability under stress [50]. Protective mechanisms triggered by hormones such as estradiol also play a role in managing stress in both men and women [51]. Given these factors, further research is required to investigate the influence of sex on stress perception and its physiological consequences.

Notably, blood pressure has been demonstrated to significantly influence obesity in adult women, whereas BMI and exercise frequency have shown significant effects on hypertension. These findings support previous research indicating a close relationship between obesity, hypertension, and metabolic disorders, particularly in women [52,53]. In adult women, physiological factors, such as blood pressure and BMI, appear to exert a greater influence on the prevalence of obesity and hypertension than psychological factors, such as perceived stress. Therefore, to better understand the development and management of obesity and hypertension in adult women, it is important to consider physiological factors, such as blood pressure and BMI, along with perceived stress [54].

Additionally, regular exercise can help maintain a healthy weight and reduce insulin resistance and systemic inflammation, all of which are implicated in hypertension [55]. Exercise can directly lower blood pressure by improving vasodilation and increasing cardiac efficiency. These findings support previous studies that demonstrated an inverse relationship between physical activity and hypertension [56]. Thus, regular exercise in adult women may prove beneficial in preventing and managing high blood pressure. However, the intensity, type, and duration of exercise suitable for the prevention and management of hypertension should be tailored to individual characteristics, warranting further research. Efforts to modify lifestyle habits to increase physical activity should also be emphasized [57]. In summary, these results also suggest potential sex-related changes in physiological systems related to blood pressure, BMI, and exercise frequency [53]. Therefore, obesity and hypertension demonstrate a correlation in adult women, underscoring the need for simultaneous blood pressure control, BMI management, and increased exercise frequency for their prevention and improvement.

The results indicating a significant impact of SBP on obesity rates in older adult women suggest a potential relationship between blood pressure and obesity in this population. These results can be attributed to physiological changes, such as hormonal changes during menopause, changes in body fat distribution, and decreased resting metabolic rates, which could increase BMI and blood pressure [58,59]. High blood pressure is a well-known risk factor for various metabolic diseases other than obesity and is particularly pronounced in older adult females, where arterial stiffness can accelerate after menopause [60]. Obesity may also have an impact on SBP in older adult women through potential interactions. Obesity, characterized by excess body fat, can contribute to an increase in blood pressure through numerous physiological changes such as activation of the sympathetic nervous system, oxidative stress, and insulin resistance [61]. Excessive adipose tissue can disrupt normal cardiovascular function, increase inflammation, and cause hormonal dysregulation, potentially contributing to hypertension [62,63]. Previous studies have suggested that obesity plays a significant role in the development of high blood pressure. However, as it is influenced by multiple factors, the relationship between obesity and high blood pressure is complex. Therefore, further research is needed to gain a deeper understanding of the interaction between obesity and SBP in older adult women.

Furthermore, the impact of perceived stress levels on obesity and hypertension appears to be less significant in the older adult population compared to adults. There are several potential reasons for the relatively small impact of perceived stress on obesity and hypertension in the older adult population. First, various physiological changes with age may have a greater impact on obesity and hypertension than perceived stress [64]. Second, a lifetime of diverse experiences may have led to the development of effective coping strategies for perceived stress, reducing its impact [65], or the timing and context of perceived stress exposure may have had different effects on health [66]. Differences in physiological responses to perceived stress, according to age and sex, may also play a role [21,67]. Nonetheless, as the results of this study demonstrated a significant impact of moderate levels of perceived stress on obesity in elderly women, detailed analyses and insights that take into account these potential reasons are required. Also, these findings suggest that effective health management should consider the effect of SBP on the prevention and management of obesity in older adult females. This means that an integrated strategy to control both blood pressure and weight could be beneficial for the health management of older adult females [68].

In addition, a significant inverse correlation was identified between breakfast intake and rates of high blood pressure in adult males. This suggests that consuming regular breakfast may help maintain circadian rhythms conducive to blood pressure control, potentially benefiting cardiovascular health. These findings support previous studies [69]. These results indicate that consistent breakfast consumption may be a beneficial strategy to prevent and manage hypertension in adult males. Sleep duration was found to be a significant factor influencing hypertension rates in older women. This corroborates previous studies that identified a positive correlation between longer sleep duration and increased incidence of hypertension [70]. The authors proposed that extended sleep duration may impair sleep quality, potentially increasing the risk of hypertension due to sleep apnea or other sleep-related disorders. However, the mechanism underlying the effects of sleep duration on hypertension has not been clearly elucidated. This indicates that an interventional approach might be necessary to understand the underlying causes of long sleep duration and to manage the rates of high blood pressure in the long sleep duration population.

This study had several limitations. First, due to its cross-sectional design, the study did not include information on how changes in perceived stress, BMI, blood pressure, and other factors evolve over time. In particular, the impact of perceived stress on various diseases can change over time; however, this study was unable to capture this. Second, the factors observed in this study do not lend themselves to clear conclusions regarding causes and effects, and it is important to be aware that unobserved variables might have influenced the results. Third, the tools and classifications used to measure the variables in this study may not fully explain these variables. Fourth, this study was conducted among Koreans, and the generalizability of the findings to populations with different cultural backgrounds may be limited. Fifth, because the number of adult and older adult participants differed significantly, the results of this study may not be comparable between groups. Sixth, only the data for the year 2015 included perceived stress, obesity, and hypertension variables. The data for the years 2017, 2019, and 2021 did not include these variables. For this reason, we are not able to update this information. Seventh, although the concept of “perceived stress” is a very broad term that encompasses multifaceted, multifactorial aspects and contributors (psychosocial, socio-economic, socio-cultural, physiological, psychological, physical, metabolic, coping behaviors (alcohol consumption, etc.)), our study did not consider these confounding variables. In the future, follow-up studies should explore such biological data in more detail to support the conclusions or hypothetical mechanisms with more confidence.

Despite these limitations, this study provides insights into the relationship between perceived stress levels, age, sex, obesity, and hypertension. The results of this study support previous research reporting the influence of sex and age on the relationship between perceived stress, BMI, obesity, and blood pressure. This suggests that there are complex interactions between sex, age, perceived stress, and metabolic disorders, including obesity and hypertension, and more detailed studies are needed to clarify these interactions [14,71]. In particular, this highlights the need for additional research to deepen our understanding of the complex relationship between perceived stress and obesity. This could be used to develop effective strategies for preventing and managing obesity and could have a positive impact on the effective management of hypertension.

## 5. Conclusions

This study identified age and sex differences in the complex relationship between perceived stress, obesity, and hypertension. Perceived stress had a significant impact on obesity in adult males, whereas blood pressure, BMI, and exercise frequency appeared to influence obesity and hypertension in adult females. In older adult females, SBP was found to influence the obesity rate, with the impact of perceived stress being less pronounced in the older adult population. These findings contribute to our understanding of the complex interplay between perceived stress and metabolic diseases, suggesting the need for further research to gain a deeper understanding of these relationships.

## Figures and Tables

**Table 1:** Baseline characteristics of adult and elderly participants.

Variables	Adults (n = 2829)	Elderly (n = 628)	*p*
Height (cm)	167.81 ± 8.74	158.86 ± 8.07	<0.001 ***
Weight (kg)	66.60 ± 11.96	60.75 ± 8.93	<0.001 ***
Body mass index (kg/m^2^)	23.53 ± 3.07	24.02 ± 2.68	<0.001 ***
Systolic blood pressure (mmHg)	123.97 ± 11.72	124.65 ± 11.52	0.192
Diastolic blood pressure (mmHg)	76.29 ± 8.80	75.23 ± 9.29	0.009 **
Perceived stress (low level)	513 (18.1%)	201 (32.0%)	<0.001 ***
Perceived stress (moderate level)	1624 (57.4%)	348 (55.4%)	
Perceived stress (high level)	692 (24.5%)	79 (12.6%)	
Exercise (frequency per week)	2.37 ± 1.90	3.71 ± 2.10	<0.001 ***
Smoker (yes/no)	511 (18.1%)	47 (7.5%)	<0.001 ***
Breakfast (yes/no)	2218 (78.6%)	580 (92.4%)	<0.001 ***
Sleep duration (hours/day)	6.64 ± 1.06	6.34 ± 1.23	<0.001 ***

Results are expressed as mean ± standard deviation or n (%). ** *p* < 0.01, *** *p* < 0.001; tested by independent *t*-test and χ^2^ test.

**Table 2:** Physical characteristics of the participants.

Variables	Adults (n = 2829)			Elderly (n = 628)		
	Males (n = 1683)	Females (n = 1146)	*p*	Males (n = 263)	Females (n = 365)	*p*
Age (years)	38.17 ± 12.71	39.39 ± 13.09	0.013 *	72.19 ± 5.25	73.60 ± 5.87	0.002 **
Height (cm)	173.11 ± 6.11	160.04 ± 5.69	<0.001 ***	165.81 ± 5.52	153.85 ± 5.49	<0.001 ***
Weight (kg)	73.15 ± 9.71	56.99 ± 7.70	<0.001 ***	66.41 ± 7.97	56.67 ± 7.20	<0.001 ***
Body mass index (kg/m^2^)	24.39 ± 2.85	22.27 ± 2.95	<0.001 ***	24.13 ± 2.43	23.94 ± 2.84	0.370
Prevalence of obesity (n, %)	642 (38.1%)	183 (16.0%)	<0.001 ***	85 (32.3%)	132 (36.2%)	0.004 **
Systolic blood pressure (mmHg)	125.52 ± 11.44	121.70 ± 11.77	<0.001 ***	125.97 ± 12.01	123.69 ± 11.07	0.014 *
Diastolic blood pressure (mmHg)	77.36 ± 8.39	74.72 ± 9.15	<0.001 ***	74.98 ± 9.45	75.40 ± 9.18	0.575
Prevalence of hypertension (n, %)	258 (15.3%)	121 (10.6%)	<0.001 ***	46 (17.5%)	46 (12.6%)	0.087
Perceived stress (low level)	304 (18.1%)	209 (18.2%)	0.290	94 (35.7%)	107 (29.3%)	0.223
Perceived stress (moderate level)	950 (56.4%)	674 (58.8%)		139 (52.9%)	209 (57.3%)	
Perceived stress (high level)	429 (25.5%)	263 (22.9%)		30 (11.4%)	49 (13.4%)	
Exercise (frequency per week)	2.46 ± 1.91	2.24 ± 1.88	0.002 **	3.92 ± 2.15	3.56 ± 2.05	0.033 *
Smoker (yes/no)	487 (28.9%)	24 (2.1%)	<0.001 ***	39 (14.8%)	8 (2.2%)	<0.001 ***
Breakfast (yes/no)	1313 (78.2%)	905 (79.1%)	0.564	242 (92.0%)	338 (92.6%)	0.785
Sleep duration (hours/day)	6.60 ± 1.05	6.71 ± 1.08	0.004 **	6.46 ± 1.24	6.26 ± 1.23	0.038 *

Results are expressed as mean ± standard deviation or n (%). * *p* < 0.05, ** *p* < 0.01, *** *p* < 0.001; tested by independent *t*-test and χ^2^ test.

**Table 3:** Correlation of obesity and hypertension according to perceived stress level in adults (19–64 years).

Variables	Perceived Stress Level			
	Low	Moderate	High	χ^2^ (*p*)
Males				
Obesity (n = 642)	93 (30.6%)	379 (39.9%)	170 (39.6%)	8.982 (0.011 *)
Hypertension (n = 258)	49 (16.1%)	136 (14.3%)	73 (17.0%)	1.838 (0.399)
Females				
Obesity (n = 183)	39 (18.7%)	102 (15.1%)	42 (16.0%)	1.479 (0.477)
Hypertension (n = 121)	23 (11.0%)	69 (10.2%)	29 (11.0%)	0.179 (0.915)

Results are expressed as n (%). * *p* < 0.05; tested by χ^2^ test.

**Table 4:** Correlation of obesity and hypertension according to perceived stress level in the elderly (65 years or above).

Variables	Perceived Stress Level			
	Low	Moderate	High	χ^2^ (*p*)
Males				
Obesity (n = 85)	29 (30.9%)	48 (34.5%)	8 (26.7%)	0.842 (0.656)
Hypertension (n = 46)	15 (16.0%)	25 (18.0%)	6 (20.0%)	0.308 (0.857)
Females				
Obesity (n = 132)	47 (43.9%)	65 (31.1%)	20 (40.8%)	5.572 (0.062)
Hypertension (n = 46)	14 (13.1%)	23 (11.0%)	9 (18.4%)	1.985 (0.371)

Results are expressed as n (%) Tested by χ^2^ test.

**Table 5:** Effect of perceived stress level on obesity and hypertension in adults (19–64 years).

Adults	Beta	Standard Error	Odds Ratio	95% Confidence Interval	*p*
Males					
Obesity (n = 642)					
Perceived stress (low)					0.017 *
Perceived stress (moderate)	0.409	0.145	1.506	(1.132~2.002)	0.005 **
Perceived stress (high)	0.379	0.166	1.461	(1.055~2.023)	0.023 *
Age (years)	0.024	0.004	1.024	(1.016~1.032)	<0.001 ***
Systolic blood pressure (mmHg)	0.000	0.005	1.000	(0.990~1.010)	0.966
Diastolic blood pressure (mmHg)	0.015	0.007	1.015	(1.001~1.029)	0.031 *
Exercise (frequency per week)	−0.041	0.028	0.960	(0.910~1.013)	0.139
Smoker (yes/no)	0.130	0.114	1.139	(0.911~1.424)	0.254
Breakfast (yes/no)	0.212	0.129	1.236	(0.961~1.591)	0.099
Sleep duration (hours/day)	−0.070	0.050	0.933	(0.846~1.029)	0.164
Hypertension (n = 258)					
Perceived stress (low)					0.427
Perceived stress (moderate)	−0.179	0.185	0.836	(0.582~1.202)	0.334
Perceived stress (high)	−0.001	0.210	0.999	(0.662~1.508)	0.997
Age (years)	−0.012	0.006	0.988	(0.977~0.999)	0.030 *
Body mass index (kg/m^2^)	0.039	0.024	1.039	(0.992~1.089)	0.103
Exercise (frequency per week)	−0.013	0.036	0.987	(0.919~1.060)	0.716
Smoker (yes/no)	0.005	0.151	1.005	(0.748~1.351)	0.973
Breakfast (yes/no)	−0.416	0.155	0.660	(0.487~0.893)	0.007 **
Sleep duration (hours/day)	0.035	0.065	1.035	(0.912~1.176)	0.594
Females					
Obesity (n = 183)					
Perceived stress (low)					0.407
Perceived stress (moderate)	−0.009	0.222	0.991	(0.641~1.532)	0.968
Perceived stress (high)	0.271	0.268	1.311	(0.776~2.216)	0.312
Age (years)	0.048	0.007	1.049	(1.034~1.064)	<0.001 ***
Systolic blood pressure (mmHg)	0.026	0.008	1.026	(1.010~1.043)	0.002 **
Diastolic blood pressure (mmHg)	0.021	0.011	1.021	(1.000~1.044)	0.055
Exercise (frequency per week)	0.077	0.046	1.080	(0.987~1.182)	0.093
Smoker (yes/no)	0.151	0.614	1.163	(0.349~3.877)	0.806
Breakfast (yes/no)	−0.347	0.212	0.707	(0.467~1.070)	0.101
Sleep duration (hours/day)	0.008	0.079	1.008	(0.864~1.176)	0.923
Hypertension (n = 121)					
Perceived stress (low)					0.857
Perceived stress (moderate)	0.013	0.267	1.013	(0.601~1.708)	0.961
Perceived stress (high)	0.140	0.313	1.150	(0.623~2.125)	0.655
Age (years)	0.020	0.009	1.020	(1.003~1.038)	0.019 *
Body mass index (kg/m^2^)	0.126	0.033	1.134	(1.063~1.210)	<0.001 ***
Exercise (frequency per week)	−0.130	0.056	0.878	(0.787~0.981)	0.021 *
Smoker (yes/no)	0.171	0.650	1.187	(0.332~4.242)	0.792
Breakfast (yes/no)	−0.185	0.239	0.831	(0.520~1.327)	0.438
Sleep duration (hours/day)	−0.023	0.090	0.977	(0.820~1.165)	0.797

* *p* < 0.05, ** *p* < 0.01, *** *p* < 0.001 vs. low perceived stress group; tested by logistic regression analysis.

**Table 6:** Effect of perceived stress level on obesity and hypertension in the elderly (65 years or above).

Elderly	Beta	Standard Error	Odds Ratio	95% Confidence Interval	*p*
Males					
Obesity (n = 85)					
Perceived stress (low)					0.608
Perceived stress (moderate)	0.120	0.308	1.127	(0.616~2.062)	0.697
Perceived stress (high)	−0.337	0.488	0.741	(0.274~1.857)	0.489
Age (years)	−0.051	0.027	0.950	(0.900~1.002)	0.061
Systolic blood pressure (mmHg)	0.016	0.012	1.016	(0.992~1.041)	0.194
Diastolic blood pressure (mmHg)	0.007	0.016	1.007	(0.976~1.038)	0.676
Exercise (frequency per week)	−0.022	0.066	0.978	(0.859~1.114)	0.737
Smoker (yes/no)	−0.138	0.411	0.871	(0.390~1.949)	0.737
Breakfast (yes/no)	−0.253	0.486	0.776	(0.299~2.012)	0.602
Sleep duration (hours/day)	−0.059	0.116	0.943	(0.750~1.185)	0.613
Hypertension (n = 46)					
Perceived stress (low)					0.857
Perceived stress (moderate)	0.125	0.381	1.134	(0.537~2.391)	0.742
Perceived stress (high)	0.303	0.557	1.354	(0.455~4.033)	0.586
Age (years)	0.015	0.032	1.015	(0.953~1.080)	0.648
Body mass index (kg/m^2^)	0.081	0.068	1.085	(0.953~1.239)	0.231
Exercise (frequency per week)	−0.074	0.080	0.929	(0.793~1.087)	0.356
Smoker (yes/no)	−0.827	0.577	0.437	(0.141~1.355)	0.152
Breakfast (yes/no)	−0.050	0.607	0.951	(0.289~3.126)	0.934
Sleep duration (hours/day)	−0.035	0.142	0.965	(0.731~1.275)	0.804
Females					
Obesity (n = 132)					
Perceived stress (low)					0.098
Perceived stress (moderate)	−0.530	0.261	0.589	(0.353~0.982)	0.042 *
Perceived stress (high)	−0.100	0.367	0.905	(0.441~1.859)	0.786
Age (years)	−0.027	0.020	0.973	(0.936~1.012)	0.170
Systolic blood pressure (mmHg)	0.029	0.012	1.029	(1.005~1.054)	0.017 *
Diastolic blood pressure (mmHg)	0.018	0.015	1.018	(0.990~1.048)	0.215
Exercise (frequency per week)	−0.006	0.057	0.994	(0.889~1.113)	0.922
Smoker (yes/no)	−0.177	0.758	0.838	(0.190~3.706)	0.816
Breakfast (yes/no)	0.474	0.453	1.607	(0.661~3.906)	0.296
Sleep duration (hours/day)	−0.063	0.094	0.939	(0.780~1.130)	0.505
Hypertension (n = 46)					
Perceived stress (low)					0.439
Perceived stress (moderate)	−0.288	0.378	0.750	(0.358~1.573)	0.447
Perceived stress (high)	0.258	0.487	1.294	(0.498~3.364)	0.597
Age (years)	−0.018	0.028	0.982	(0.929~1.037)	0.515
Body mass index (kg/m^2^)	0.074	0.056	1.077	(0.966~1.200)	0.183
Exercise (frequency per week)	−0.110	0.081	0.896	(0.764~1.050)	0.176
Smoker (yes/no)	−0.122	1.133	0.886	(0.096~8.164)	0.915
Breakfast (yes/no)	0.082	0.603	1.086	(0.333~3.538)	0.892
Sleep duration (hours/day)	0.265	0.130	1.303	(1.011~1.680)	0.041 *

* *p* < 0.05 vs. low perceived stress group, tested using logistic regression analysis.

## Data Availability

The data presented in this study are available upon request from the corresponding author. The data are not publicly available because of the protection of personal information.
